# Case Report: Iatrogenic dyspnea and respiratory failure in a multiple myeloma patient treated with carfilzomib

**DOI:** 10.3389/fonc.2025.1701639

**Published:** 2025-11-12

**Authors:** Giuseppe Bertuglia, Mattia Verardo, Matilde Scaldaferri, Mauro Mangiapia, Benedetto Bruno, Alessandra Larocca

**Affiliations:** 1 Division of Hematology, Azienda Ospedaliero-Universitaria Città della Salute e della Scienza di Torino, University of Torino, Torino, Italy; 2 Department of Molecular Biotechnology and Health Sciences, University of Torino, Torino, Italy; 3 Pneumology Unit, Department of cardiovascular and thoracic diseases, Azienda Ospedaliero Universitaria Città della Salute e della Scienza di Torino, Torino, Italy; 4 Hospital Pharmacy, Azienda Ospedaliero Universitaria Città della Salute e della Scienza di Torino, Turin, Italy

**Keywords:** multiple myeloma, carfilzomib, dyspnea, respiratory failure, adverse event

## Abstract

Carfilzomib, a second-generation proteasome inhibitor, is approved for the treatment of relapsed or refractory multiple myeloma, with cardiovascular toxicity being the most common concern. Nevertheless, pulmonary complications have been reported and may complicate differential diagnosis. We describe the case of a 79-year-old man with multiple myeloma who developed two episodes of acute respiratory failure temporally related to carfilzomib administration. After six cycles of therapy, the patient presented with dyspnea, hypoxemia, and bronchospasm requiring hospitalization. Extensive investigations excluded infection, pulmonary embolism, and cardiac dysfunction, and symptoms resolved with supportive therapy. Carfilzomib was resumed at reduced dose, but a second episode of respiratory failure occurred shortly after re-exposure, leading to definitive discontinuation. Pulmonary function testing demonstrated recovery to baseline values after drug withdrawal. This case underlines the need for awareness of non-cardiogenic respiratory adverse events in patients treated with carfilzomib, especially those with pre-existing pulmonary conditions. Careful monitoring and appropriate diagnostic work-up are essential, and drug re-challenge should be avoided in case of severe respiratory events. Recognition of these complications may improve patient safety and guide therapeutic decisions in multiple myeloma.

## Introduction

Carfilzomib is a second-generation proteasome inhibitor (PI) which is approved for the treatment of relapsed refractory multiple myeloma (MM). Whatever cardiovascular effects (blood pressure disorders and heart failure) were more feared and a careful cardiovascular work-up is suggested before carfilzomib administration, dyspnea and pulmonary complications were described. We report a case of respiratory failure related to carfilzomib, with a second episode relapsing despite of reduction of dose.

## Case description

A 79-year-old man was treated in third line with carfilzomib and dexamethasone (Kd) for IgG-L multiple myeloma. His medical history included hypertension treated with ramipril, right bundle branch block and left anterior fascicular block, and hypertriglyceridemia in treatment with gemfibrozil. He had previously received lenalidomide and dexamethasone plus radiotherapy for a plasmacytoma located in the T11 thoracic vertebra as first-line therapy, and daratumumab-bortezomib-dexamethasone as second-line treatment. Due to his cardiovascular risk factors, a thorough pre-treatment work-up was performed before carfilzomib administration: ejection fraction of left ventricular was normal (63%) without any issue on valves and lipids levels and blood pressure were under control with drug therapy as well.

Carfilzomib was administered as a 30-minute intravenous infusion at a dose of 20 mg/m² on days 1 and 2 of cycle 1, followed by 56 mg/m² on subsequent cycles, given on days 1, 2, 8, 9, 15, and 16 of a 28-day cycle. Dexamethasone (20 mg, oral) was administered on days 1, 2, 8, 9, 15, 16, 22, and 23. He received aspirin for thromboembolic prophylaxis.

Paraprotein fell down from 18 to 3 g/L after six cycles, reaching partial response as best response. Nevertheless, he complained of mild shortness of breath on exertion that did not limit his daily activities. A diagnostic evaluation was carried out prior to cycle seven due to the onset of dyspnea: his blood pressure diary showed in range blood pressure records; echocardiography excluded heart failure or any concern compared to the other one performed before treatment; daily oxygen saturation was consistently around 95-98%; thoracic consecutive X-rays were unremarkable. However, pulmonary function testing demonstrated severe airflow obstruction: Forced Vital Capacity (FVC) 1.06 (40%), Forced Expiratory Volume in 1 second (FEV1) 0.67 (37%), Total Lung Capacity (TLC) 3.14 (60%), Residual Volume (RV) 2.06 (82%), Residual Volume/Total Lung Capacity (RV/TLC) 147%, Diffusing Capacity for Carbon Monoxide (DLCO) 48%. Compared to a previous evaluation two years earlier [FVC 1.62 (64%), FEV1 1.18 (61%), TLC 4.00 (75%), RV 2.46 (89%), RV/TLC 120%, DLCO 85%], a progressive decline was observed. No identifiable cause was found, raising the suspicion of previously unrecognized restrictive lung disease with associated air trapping. These abnormalities were considered likely due to thoracic kyphosis, caused by vertebral collapse occurred at diagnosis, and age-related muscle hypotonia, and no specific drug was recommended by the consultant pulmonologist.

On day 8 of cycle seven, the patient presented at day clinic with significant dyspnea, tachypnoea (28 breaths per minute) and oxygen saturation of 88%. His temperature was 37.7 °C without either cough or chest pain. On physical examination, diffuse wheezing and coarse breath sounds were noted. Arterial blood gas analysis revealed type 1 respiratory failure (pH 7.36, pCO2 41, pO2 43); thus, he was hospitalized for further investigations. Blood tests showed slightly raised C-reactive protein (CRP) 34.2 mg/L and normal levels of serum N-terminal prohormone brain natriuretic peptide (NT-proBNP), troponin and fibrinogen without any further concerns. CT pulmonary angiography (CTPA) ruled out both thromboembolism and pneumonia. A transthoracic echocardiogram was also performed, showing no cardiac abnormalities suggestive of heart failure and no significant changes compared to the previous assessment. He received oxygen therapy (Venturi mask 35%), low-dose steroids, ipratropium bromide/salbutamol as nebulization and empiric antibiotic therapy with levofloxacin. The patient gradually improved and oxygen was discontinued in the following days, with a complete resolution of the symptoms. This event ultimately appeared to have been triggered by an infection, without a clear correlation to carfilzomib.

Given the good response to this third-line therapy, and the resolution of the event, carfilzomib was re-started with reduced dose of 45 mg/m² (80% of the initial dose) after the discharge.

The first dose was administered a few days after discharge, apparently without complications. On day 2, the patient presented at the day clinic with tachypnoea (38 breaths per minute), tachycardia (115 beats per minute), hypotension (95/40 mmHg) and fever (37.7 °C). On the physical examination, diffuse wheezes were noted. Arterial blood gas analysis again showed type 1 respiratory failure (pH 7.40, pCO2 42, pO2 48), and he was hospitalized. Blood tests were superimposable to the previous ones (CRP 33.6, normal levels of NT-proBNP, troponin and fibrinogen). Swabs for influenza virus, Respiratory Syncytial Virus, rhinovirus, and SARS-CoV-2 were also negative. Thoracic CT excluded again thromboembolism and pneumonia. Oxygen therapy (Venturi mask 35%), ipratropium bromide/salbutamol and low-dose steroids were administered. In the following days, he gradually improved and he was discharged without oxygen therapy.

In both episodes, the temporal association between symptom onset and improvement after discontinuation of carfilzomib, along with the exclusion of other potential causes, suggested that the patient’s symptoms were likely secondary to carfilzomib.

The patient remained off-therapy for three months without any respiratory symptoms. Unfortunately, his paraprotein increased from 3 g/L to 10 g/L suggesting multiple myeloma relapse, thus treatment with elotuzumab, pomalidomide, and dexamethasone was started. He achieved a very good partial response and remained on treatment for one year without other respiratory concerns.

Twelve months after the acute episodes of respiratory failure and bronchospasm, pulmonary function tests demonstrated significant improvement compared to those performed during carfilzomib therapy, with restoration of lung volumes to baseline levels: FVC 1.65 (67%), FEV1 1.01 (56%), TLC 5.27 (100%), and DLCO 69%. A summary of pulmonary function parameters over time is provided in **Table 1**.

**Table 1:** Pulmonary function test parameters two years before, during, and one year after carfilzomib treatment.

Pulmonary function test parameters	Normal value	Before treatment	During treatment	After treatment
FVC	≥ 80%*	1.62 (64%)	1.06 (40%)	1.65 (67%),
FEV_1_	≥ 80%*	1.18 (61%)	0.67 (37%)	1.01 (56%)
FEV_1_/FVC Ratio	≥ 70%	68 (93%)	69 (94%)	61 (84%)
TLC	80–120%*	4.00 (75%)	3.14 (60%)	5.27 (100%)
RV	80–120% *	2.46 (89%)	2.06 (82%)	3.88 (152%)
RV/TLC	20–35%	54 (120%)	65 (147%)	74 (163%)
DLCO	≥ 80%*	16 (85%)	8 (48%)	12 (69%)

The table reports individual values of key pulmonary function test (PFT) parameters—including FVC, FEV_1_, FEV_1_/FVC ratio, TLC, RV, RV/TLC, and DLCO—measured at three timepoints: baseline (prior to treatment), during treatment, and after completion or discontinuation of carfilzomib. Values are presented as absolute numbers and percentages predicted.

FVC, Forced Vital Capacity; FEV1, Forced Expiratory Volume in 1 second; TLC, Total Lung Capacity; RV, Residual Volume; DLCO, Diffusing Capacity for Carbon Monoxide.

*Of predicted value.

## Discussion

Carfilzomib is an irreversible proteasome inhibitor that targets the chymotrypsin-like catalytic subunit (β5) of the 20S proteasome (1). It is approved for the treatment of relapsed or refractory multiple myeloma in patients who have received at least one prior line of therapy, alone with dexamethasone (2) or in combination with other drugs (3–5).

Although the primary concern with carfilzomib is its cardiovascular toxicity (6), it is equally important to recognize the potential for pulmonary adverse events.

Indeed, dyspnea has been reported as an adverse event in clinical trials that led to the approval of carfilzomib, even in combination with dexamethasone alone (any grade: 21-32%; ≥3 grade: 1-6%) (2) or in combination with immunomodulatory drugs (any grade: 11-19%; ≥3 grade: 1-3%) (3, 7, 8) or anti-CD38 monoclonal antibodies (any grade: 19%-28%; ≥3 grade: 3-5%) (4, 5).

In an analysis of four phase II clinical studies including single agent carfilzomib (9), the most commonly reported pulmonary adverse events were dyspnea (42%), mainly low grade, and cough (26%), which resulted in dose reduction in 2% and treatment discontinuation in an additional 2% of patients. Dyspnea resolved in 67.9% of patients. Additionally, respiratory tract infections and pneumonia were reported in 19% and 13%, respectively. Other less common pulmonary adverse events included pleural effusion (4%), pulmonary hypertension (2%), pulmonary embolism (1%), hemoptysis (1%), and pneumonitis (<1%). No interstitial lung disease or pulmonary fibrosis was reported.

There have been sporadic cases of more serious pulmonary adverse events. Fatal pulmonary toxicity manifested as acute respiratory distress syndrome (ARDS) and diffuse alveolar hemorrhage within one week of receiving the first dose of carfilzomib, and carfilzomib-related severe pneumonitis have been reported in literature (10–12).

A study based on the Surveillance, Epidemiology and End Results (SEER)–Medicare linked database (13), described the cardiopulmonary effects of carfilzomib in a population of 635 patients. Specifically, ~28% of patients included in the study developed dyspnea and 15% developed cough. Pneumonia and respiratory tract infections were each listed in 15% and 14% of patients, respectively. New venous thromboembolic events were documented in 6% of patients and 1% of the patients were noted to develop pulmonary hypertension.

To explore whether dyspnea has previously been associated with carfilzomib in the setting of real-world use, the FDA Adverse Event Reporting System (FAERS) (14) was consulted. As of March 2025, 909 reports of dyspnea out of 2,890 cases of Respiratory, Thoracic and Mediastinal disorders have been recorded in association with carfilzomib, representing approximately 4.2% of 21,778 total adverse event reports for this drug. Notably, all these cases were classified as non-cardiogenic in nature. Although spontaneous reporting systems such as FAERS are subject to limitations, particularly under-reporting, lack of verification, the absence of data on monotherapy or combination use and on comorbidities, and the absence of exposure data, these findings provide supportive evidence for a possible association between carfilzomib and dyspnea, as observed in the present case.

The presence of dyspnea, which is a relevant symptom both in pulmonary and in cardiac conditions such as heart failure, can complicate the clinical picture. Moreover, patients with preexisting pulmonary conditions may be at increased risk of developing respiratory complications while on carfilzomib. In particular, a history of chronic obstructive pulmonary disease (COPD) was independently associated with a 40% increased hazard (HR 1.4) of developing such complications (13). This makes the differential diagnosis more challenging (drug-related toxicity, cardiovascular events, recurrence of COPD, and pulmonary infection).

In our patients, spirometry was not performed prior to the start of treatment, so the presence of pre-existing restrictive lung disease cannot be determined. The clinical presentation may partly resemble asthma flare-ups with a respiratory type 1 respiratory failure, but the patient required treatment with beta-2 agonists and muscarinic antagonists only during hospitalization and had no respiratory symptoms before starting or after discontinuing the therapy.

The exact mechanisms underlying carfilzomib-induced pulmonary toxicity remain unclear. Current hypotheses suggest that carfilzomib may induce an inflammatory state through the elevation of pro-inflammatory pathways involving TNF-α and NF-kB, along with the downregulation of anti-inflammatory mediators like IL-10 and reduction in glutathione levels, which serves as a natural intracellular antioxidant (15). Effects on the cardiovascular system is likely related to interference with nitric oxide synthase (NOS) activity and nitric oxide (NO) levels. Loss of NO bioavailability leads to impaired vasodilation and endothelial dysfunction leading to hypertension, coronary artery disease, chronic heart failure, peripheral artery disease, and chronic renal failure, while NO upregulation is associated to improvement of carfilzomib related toxicity (16). Similarly, carfilzomib is associated to thrombotic microangiopathy likely due to excessive complement activation, reduction of vascular endothelial growth factor (VEGF) levels via NF-κB inhibition and endothelial injury (17). The hypothesis that carfilzomib may damage also lung vessels make reasonable as pulmonary hypertension is also reported (18). In summary, the reduction of NO combined with the activation of inflammatory pathways can cause damage not only to the heart and kidneys, but also to the lungs. Several studies have correlated the dysregulation of the endothelial nitric oxide pathway with airway inflammation (19, 20), leading to increased bronchial inflammatory states and episodes of bronchospasm, as described in the lungs of mice exposed to carfilzomib (15).

Although it has been hypothesized that carfilzomib may be toxic through a cumulative dose-dependent mechanism (21), studies are conflicting (22). In our patient, dyspnea was well-tolerated during the first cycles, but carfilzomib dose reduction did not prove to be effective, and therefore, re-administration should be carefully evaluated and ultimately avoided, especially in the presence of life-threatening events and other treatment options.

Given the potential overlap of symptoms, monitoring, thorough clinical evaluation, and appropriate diagnostic testing, such as echocardiography, pulmonary function tests, and imaging, are essential to ensure accurate diagnosis and prompt intervention, mainly in patients with underline pulmonary disease. In clinical practice, NT-proBNP levels, rather than troponin levels, can be useful in distinguishing the cause of dyspnea, as elevated NT-proBNP has been observed in all cases of dyspnea related to cardiotoxicity (22).

## Conclusion

In conclusion, while cardiovascular toxicity remains the most prominent concern with carfilzomib, it is imperative to remain vigilant about its pulmonary side effects, as showed in this case report. Although the gaining of new drugs in multiple myeloma armamentarium such as bispecific antibodies and CAR-T, carfilzomib remains a backbone of the treatment, and his role is still under investigation in prior line and high-risk patients (23–25). Understanding the full spectrum of carfilzomib’s toxicities and recognizing the shared symptoms between cardiac and pulmonary complications can help guide more precise clinical decision-making and improve patient outcomes.

## Data Availability

The original contributions presented in the study are included in the article/supplementary material. Further inquiries can be directed to the corresponding author.

## References

[1] Jayaweera SPE Wanigasinghe Kanakanamge SP Rajalingam D Silva GN . Carfilzomib: A promising proteasome inhibitor for the treatment of relapsed and refractory multiple myeloma. Front Oncol. (2021) 11:740796. doi: 10.3389/fonc.2021.740796, PMID: 34858819 PMC8631731

[2] Dimopoulos MA Goldschmidt H Niesvizky R Joshua D Chng WJ Oriol A . Carfilzomib or bortezomib in relapsed or refractory multiple myeloma (ENDEAVOR): an interim overall survival analysis of an open-label, randomized, phase 3 trial. Lancet Oncol. (2017) 18:1327–37. doi: 10.1016/S1470-2045(17)30578-8, PMID: 28843768

[3] Mateos MV Goldschmidt H San-Miguel J Mikhael J DeCosta L Zhou L . Carfilzomib in relapsed or refractory multiple myeloma patients with early or late relapse following prior therapy: A subgroup analysis of the randomized phase 3 ASPIRE and ENDEAVOR trials. Hematol Oncol. (2018) 36:463–70. doi: 10.1002/hon.2499, PMID: 29446103

[4] Usmani SZ Quach H Mateos MV Landgren O Leleu X Siegel D . Final analysis of carfilzomib, dexamethasone, and daratumumab vs carfilzomib and dexamethasone in the CANDOR study. Blood Adv. (2023) 7:3739–48. doi: 10.1182/bloodadvances.2023010026, PMID: 37163358 PMC10368773

[5] Moreau P Dimopoulos MA Mikhael J Yong K Capra M Facon T . Isatuximab, carfilzomib, and dexamethasone in relapsed multiple myeloma (IKEMA): a multicenter, open-label, randomized phase 3 trial. Lancet. (2021) 397:2361–71. doi: 10.1016/S0140-6736(21)00592-4, PMID: 34097854

[6] Bringhen S Milan A D'Agostino M Ferri C Wäsch R Gay F . Prevention, monitoring and treatment of cardiovascular adverse events in myeloma patients receiving carfilzomib A consensus paper by the European Myeloma Network and the Italian Society of Arterial Hypertension. J Intern Med. (2019) 286:63–74. doi: 10.1111/joim.12882, PMID: 30725503

[7] Gay F Musto P Rota-Scalabrini D Bertamini L Belotti A Galli M . Carfilzomib with cyclophosphamide and dexamethasone or lenalidomide and dexamethasone plus autologous transplantation or carfilzomib plus lenalidomide and dexamethasone, followed by maintenance with carfilzomib plus lenalidomide or lenalidomide alone for patients with newly diagnosed multiple myeloma (FORTE): a randomized, open-label, phase 2 trial. Lancet Oncol. (2021) 22:1705–20. doi: 10.1016/S1470-2045(21)00535-0, PMID: 34774221

[8] Perrot A Delimpasi S Spanoudakis E Frølund U Belotti A . An open-label phase 2 study treating patients with first or second relapse of multiple myeloma with carfilzomib, pomalidomide, and dexamethasone (KPd): SELECT study. Leuk Lymphoma. (2024) 65:833–42. doi: 10.1080/10428194.2024.2322030, PMID: 38497533

[9] Wang M Cheng J . Overview and management of cardiac and pulmonary adverse events in patients with relapsed and/or refractory multiple myeloma treated with single-agent carfilzomib. Oncol (Williston Park). (2013) 27:24–30. 25184233

[10] Bhagavath A Geyer A . Carfilzomib pulmonary toxicity. Chest. (2016) 150:492A. doi: 10.1016/j.chest.2016.08.506

[11] Lataifeh AR Nusair A . Fatal pulmonary toxicity due to carfilzomib (Kyprolis™). J Oncol Pharm Pract. (2016) 22:720–4. doi: 10.1177/1078155215588630, PMID: 26044587

[12] Sridhar A Yohannan B Kachira JJ Bhama S . Severe pulmonary toxicity related to carfilzomib use: a rare but serious side effect. BMJ Case Rep. (2022) 15:e251173. doi: 10.1136/bcr-2022-251173, PMID: 35787495 PMC9255386

[13] Fakhri B Fiala MA Shah N Vij R Wildes TM . Measuring cardiopulmonary complications of carfilzomib treatment and associated risk factors using the SEER-Medicare database. Cancer. (2020) 126:808–13. doi: 10.1002/cncr.32601, PMID: 31721140 PMC6992490

[14] FDA adverse event reporting system (FAERS) public dashboard . Available online at: https://www.fda.gov/drugs/fdas-adverse-event-reporting-system-faers/fda-adverse-event-reporting-system-faers-public-dashboard (Accessed March 31, 2025).

[15] Habib CN Ali AE Anber NH George MY . Lactoferrin ameliorates carfilzomib-induced renal and pulmonary deficits: Insights to the inflammasome NLRP3/NF-κB and PI3K/Akt/GSK-3β/MAPK axes. Life Sci. (2023) 335:122245. doi: 10.1016/j.lfs.2023.122245, PMID: 37926296

[16] Al-Harbi NA Imam F Al-Harbi MM Qamar W Aljerian K Anwer MK . Apremilast ameliorates carfilzomib-induced pulmonary inflammation and vascular injuries. Int Immunopharmacol. (2019) 66:260–6. doi: 10.1016/j.intimp.2018.11.023, PMID: 30500623

[17] Ponraj R Bryant A Dunlop L Range H Cobrador C Ling S . Carfilzomib-induced thrombotic microangiopathy (TMA): an under-recognized spectrum of disease from microangiopathic hemolysis to subclinical TMA. Blood Cancer J. (2023) 13:113. doi: 10.1038/s41408-023-00885-9, PMID: 37495597 PMC10371986

[18] Yang JZ Buckstaff T Narezkina A Fernandes TM . Carfilzomib-associated pulmonary arterial hypertension in multiple myeloma. Pulm Circ. (2021) 11:1–3. doi: 10.1177/20458940211049300, PMID: 34603687 PMC8485285

[19] Csoma B Bikov A Nagy L Tóth B Tábi T Szűcs G . Dysregulation of the endothelial nitric oxide pathway is associated with airway inflammation in COPD. Respir Res. (2019) 20:156. doi: 10.1186/s12931-019-1133-8, PMID: 31311549 PMC6636120

[20] Phillips D Brotto A Ross B Bryan T Wong E Van Diepen S . The effect of inhaled nitric oxide on dyspnea and exercise capacity in mild COPD. Clin Respir physiology Exercise Funct Imaging. (2019), OA3572. doi: 10.1183/13993003.congress-2019.OA3572

[21] Chari A Hajje D . Case series discussion of cardiac and vascular events following carfilzomib treatment: possible mechanism, screening, and monitoring. BMC Cancer. (2014) 14:915. doi: 10.1186/1471-2407-14-915, PMID: 25471129 PMC4289164

[22] Dimopoulos MA Roussou M Gavriatopoulou M Psimenou E Ziogas D Eleutherakis-Papaiakovou E . Cardiac and renal complications of carfilzomib in patients with multiple myeloma. Blood Adv. (2017) 1:449–54. doi: 10.1182/bloodadvances.2016003269, PMID: 29296960 PMC5738981

[23] Costa LJ Chhabra S Medvedova E Dholaria BR Schmidt TM Godby KN . Daratumumab, carfilzomib, lenalidomide, and dexamethasone with minimal residual disease response-adapted therapy in newly diagnosed multiple myeloma. J Clin Oncol. (2022) 40:2901–12. doi: 10.1200/JCO.21.01935, PMID: 34898239

[24] Gay F Roeloffzen W Dimopoulos MA Rosiñol L van der Klift M Mina R . Results of the phase III randomized iskia trial: isatuximab-carfilzomib-lenalidomide-dexamethasone vs carfilzomib-lenalidomide-dexamethasone as pre-transplant induction and post-transplant consolidation in newly diagnosed multiple myeloma patients. Blood. (2023) 142:4–4. doi: 10.1182/blood-2023-177546 37410508

[25] Mai EK Bertsch U Pozek E Fenk R Besemer B Hanoun C . satuximab, lenalidomide, bortezomib, and dexamethasone induction therapy for transplant-eligible newly diagnosed multiple myeloma: final part 1 analysis of the GMMG-HD7 trial. J Clin Oncol. (2024) 43:1279–88. doi: 10.1200/JCO-24-02266, PMID: 39652594 PMC11974620

